# Has GWAS lost its status as a paragon of open science?

**DOI:** 10.1371/journal.pbio.3001242

**Published:** 2021-05-03

**Authors:** Callie Burt, Marcus Munafò

**Affiliations:** 1 Department of Criminal Justice & Criminology, Georgia State University, Atlanta, United States of America; 2 Center for Research on Interpersonal Violence, Georgia State University, Atlanta, United States of America; 3 MRC Integrative Epidemiology Unit at the University of Bristol, Bristol, United Kingdom; 4 School of Psychological Science, University of Bristol, Bristol, United Kingdom

## Abstract

Genomic research led the way in open science – a tradition continued by genome-wide association studies – through the sharing of materials, results and data. Coordinated quality control procedures also contributed to robust findings. But this Perspective article wonders whether these standards are now slipping back.

## Teaser

Genomic research led the way in open science, a tradition continued by genome-wide association studies (GWAS)—through the sharing of materials, results, and data. Coordinated quality control procedures also contributed to robust findings. However, recent years have seen declines in GWAS transparency. Here, we assess some shifts away from open science practices with the aim of stimulating a discussion of these issues.

The Human Genome Project (HGP) led the way in open science—in particular, data sharing. In 1996, HGP scientists established the “Bermuda Principles,” which specified that DNA sequence data should be released in publicly accessible databases within 24 hours of generation. The following year, data quality standards were developed—the “Bermuda Sequence-Quality Standards.” These Bermuda agreements (see https://web.ornl.gov/sci/techresources/Human_Genome/research/bermuda.shtml) were key to the multinational collaborative work behind the HGP’s remarkable successes, producing a global knowledge resource that has stimulated major scientific advances. Following completion of the HGP, these open science principles were applied to other genomics projects. Scientists and funders recognized the value of data sharing, coordination, and transparency in advancing knowledge, scientific credibility, and improvements in human health. Many data sharing policies reflect the ethos of these principles, and many areas of human genomics continue lead the way in open science.

Genome-wide association studies (GWAS) continued these open science trends. The GWAS era arose following the widespread recognition of low statistical power and questionable methodological practices in candidate gene association studies, which were plagued by low reproducibility. The need to collaborate at scale to achieve the large sample sizes required to detect small effect sizes, while correcting for multiple testing, necessitated coordinated data analysis plans and, in turn, harmonized datasets and code. These were shared within consortia, making it a small step to sharing materials, results, and data publicly (albeit typically summary results, rather than individual level data). Another benefit of this collaborative approach (particularly when handling complex datasets) was a focus on coordinated quality control procedures. For these reasons, human genomics in general, and GWAS, in particular, are often held up as an exemplar of reproducible science. Does the GWAS field still live up to these standards, or is it slipping back?

GWAS is now a mainstream technique, and increasingly only one part of a study, rather than the study itself. Studies that include a GWAS now often include functional work, analysis of causal pathways, polygenic risk score analyses, and so on. But this greater breadth risks coming at a cost; often the details of the GWAS itself are relegated to a supplement, which reviewers may scrutinize less carefully [1], while the need to recruit reviewers to evaluate these other elements comes at the expense of having multiple experts inspect the GWAS itself, if this is no longer the sole focus.

There is evidence that this has been accompanied by inconsistency in standards. We have seen imputation quality scores as high as r^2^ > .9 to an imputation accuracy score of < .1 [2]. GWAS may employ different thresholds across cohorts and analyses within the same study. While what is acceptable will depend on the specific nature of the study, these different thresholds may have a substantial impact on results. However, because imputed SNPs that pass the threshold are not treated any differently from measured SNPs, and imputation quality scores are not included in GWAS, we have no way of knowing whether this is a problem. Different software packages and bioinformatic pipelines are employed, with assumptions that may not be articulated. Even commonly adopted minimum thresholds for what constitutes “sufficient LD” for the purposes of identifying SNP “independence” (e.g., r^2^ <. 1) vary across studies, as well different analyses within studies.

Employing different thresholds may be warranted, but methodological decisions should be clearly documented and justified. In our view, simply relying on honesty, and assuming no mistakes, is not the best way forward in modern science, where the incentives to produce noteworthy findings can be substantial. Transparency can serve a quality control function [3]. The extraordinary complexity and density of many current studies including a GWAS means methodological details can be relegated to extensive supplements. If these are not scrutinised fully, this may impact on the robustness and reproducibility of GWAS results, with downstream effects such as overinterpretation of noise (e.g., post-GWAS analyses, such as gene prediction, tissue specific expression). Further, many bioinformatic pipelines use existing associations and functional annotations to link to new findings.

Alongside this increase in complexity, there has also been a shift away from open science practices. Efforts to achieve ever-greater sample sizes, coupled with the finite number of high-quality large cohorts with genetic information available, have encouraged researchers to increasingly partner with private companies that can offer large amounts of data. These companies have a direct interest in using GWAS results for profit and thus have a motivation to contribute data. But the results are commercially sensitive. One consequence is that these private-public research partnerships proceed largely on the terms of the private companies. These terms commonly include no access to individual data (analyzed with “in house pipelines”), no sharing of data, and sharing of only partial results.

Many recently published GWAS using 23andMe data include partial results, no code, and no data [e.g., 4,5]. This is despite the fact that most of these studies are meta-analyses, and the data consist of summary statistics, rather than the primary, individual-level data, and therefore do not include sensitive, individually identifiable information. Furthermore, such closed data practices often contravene explicit journal and funding agency data sharing policies. The result is that researchers’ ability to replicate and build on these studies is limited.

Commercial datasets are also often highly unrepresentative. The problem of lack of representativeness is not unique to commercial datasets—for example, UK Biobank achieved only an approximately 5% recruitment rate, with evidence of “healthy volunteer” selection bias into that study [6]. But selection into commercial datasets can be particularly pronounced. The widely used 23andMe data is composed primarily of individuals from the USA who can afford to investigate their DNA. Participants therefore tend to be European-ancestry, more highly educated, more affluent, and in better health. Furthermore, these data can make up a considerable proportion of the total sample in a GWAS—in some cases over 50% of the total sample [5]. These highly selected samples may bias results [7]. This concern is especially acute for socially patterned phenotypes such educational attainment, income, health behaviours, and mental health (which are often minimally phenotyped via brief participant self-report). The quest for ever-larger sample sizes seems to have come at the expense of the transparency and data sharing that characterized the field in the past. Collaborations between academia and industry can be powerful, and consortium efforts have been critical to the success of GWAS efforts, but we should always ask: At what cost? The question of whether this trade-off is a net positive deserves attention. We would encourage an open discussion of the costs and benefits of these trade-offs by the research community.

Despite having led the way in open science and reproducibility, GWAS has become more opaque. Perhaps the method is being taken for granted, given its track record of generating reproducible findings; but reproducible science requires enforcing existing standards as well as continued review and refinement [8]. The lesson is that no methodology stands still, and as particularly complex methodologies evolve—whether it be GWAS, fMRI, etc.—we should continue to examine how these methodologies are applied, and how robust the findings they generate are. If GWAS wants to remain a paragon of open science, it cannot be open only when convenient. Otherwise, hard-won gains in openness and reproducibility can be gradually eroded often at a significant cost to scientific credibility.
